# Data collection and storage in long-term ecological and evolutionary studies: The Mongoose 2000 system

**DOI:** 10.1371/journal.pone.0190740

**Published:** 2018-01-09

**Authors:** Harry H. Marshall, David J. Griffiths, Francis Mwanguhya, Robert Businge, Amber G. F. Griffiths, Solomon Kyabulima, Kenneth Mwesige, Jennifer L. Sanderson, Faye J. Thompson, Emma I. K. Vitikainen, Michael A. Cant

**Affiliations:** 1 Centre for Ecology and Conservation, University of Exeter, Penryn Campus, Cornwall, United Kingdom; 2 Centre for Research in Ecology, Evolution and Behaviour, University of Roehampton, London, United Kingdom; 3 FoAM Kernow, Jubilee Warehouse, Penryn, Cornwall, United Kingdom; 4 Banded Mongoose Research Project, Queen Elizabeth National Park, Kasese District, Rubirizi, Uganda; 5 Department of Biosciences, University of Helsinki, Helinski, Finland; University of Adelaide, AUSTRALIA

## Abstract

Studying ecological and evolutionary processes in the natural world often requires research projects to follow multiple individuals in the wild over many years. These projects have provided significant advances but may also be hampered by needing to accurately and efficiently collect and store multiple streams of the data from multiple individuals concurrently. The increase in the availability and sophistication of portable computers (smartphones and tablets) and the applications that run on them has the potential to address many of these data collection and storage issues. In this paper we describe the challenges faced by one such long-term, individual-based research project: the Banded Mongoose Research Project in Uganda. We describe a system we have developed called Mongoose 2000 that utilises the potential of apps and portable computers to meet these challenges. We discuss the benefits and limitations of employing such a system in a long-term research project. The app and source code for the Mongoose 2000 system are freely available and we detail how it might be used to aid data collection and storage in other long-term individual-based projects.

## Introduction

The natural environment is a rich and interconnected world. In many instances answering ecological and evolutionary questions about it requires years of study following individual organisms throughout their lives. Long-term, individual-based research projects fulfil this need and have played a vital role in the study of ecological and evolutionary processes [1], and in anticipating how environmental change will impact the natural world in the future [2,3].

The long-term and individual-based nature of these research projects is the source of their success but also presents a major logistical challenge, requiring the collection of multiple streams of data from multiple individuals at multiple points in time. For example, guided by Tinbergen’s four questions [4], behavioural ecologists often seek to understand how proximate mechanisms, such as hormonal effects, and ultimate causes, such as effects on reproduction and survival, shape variation in behaviour within and between individual animals. For each individual in such a study this requires recording their behaviour at a given point in time, collecting a tissue sample from them (e.g. faeces) at the same time for later physiological analysis, and monitoring their survival and reproduction over a longer time period [5–7]. These data are almost always collected in field locations that are unsuitable for standard computing equipment. As a result these data usually need to be recorded in the field and then manually transferred to a central database at a later date in an often error-prone and time-consuming process [8]. At best, this requirement takes up skilled fieldworkers’ valuable time and introduces noise into the project’s database. At worst, this may create biased errors which may then lead to erroneous findings.

Over the last decade there has been a dramatic rise in the availability and sophistication of cheap, portable computers (e.g. smartphones, tablet computers). These portable computers and the programs (or ‘apps’) that run on them provide a huge opportunity for ecological scientists [9,10]. They have the potential to collect and store data from multiple streams, in a pre-determined, structured manner, with built-in error checking. Data streams may come from a range of on-board sensors (e.g. camera, GPS, climate), or be generated manually (e.g. from a fieldworker recording an observation, result, or note). Apps and portable computers have become widely used in disciplines such as engineering and medicine [9]. However, their use in ecological studies has generally been limited to data collection by members of the public in citizen science projects [11,12] rather than by skilled fieldworkers (but see exceptions such as EpiCollect [8]; Ant-App [13]; and DORIS [9]).

Data collection on long-term research projects is usually conducted by skilled fieldworkers, trained in the principles of robust data collection and the biology of the particular study system. Apps designed for use by such skilled fieldworkers can incorporate a broader and more complex range of inputs and interfaces than would be optimal if the app was designed for an untrained user. There is huge potential to create bespoke apps to maximise the return on the skills of such fieldworkers, and to improve the efficient and accurate collection and storage of data on field projects. In this paper we illustrate this potential benefit with a case study of a long-term, individual-based research project, the Banded Mongoose Research Project, and a system utilising apps and portable computers developed to meet this project’s data collection and storage challenges: Mongoose 2000.

## The banded mongoose research project

**Fig 1:**
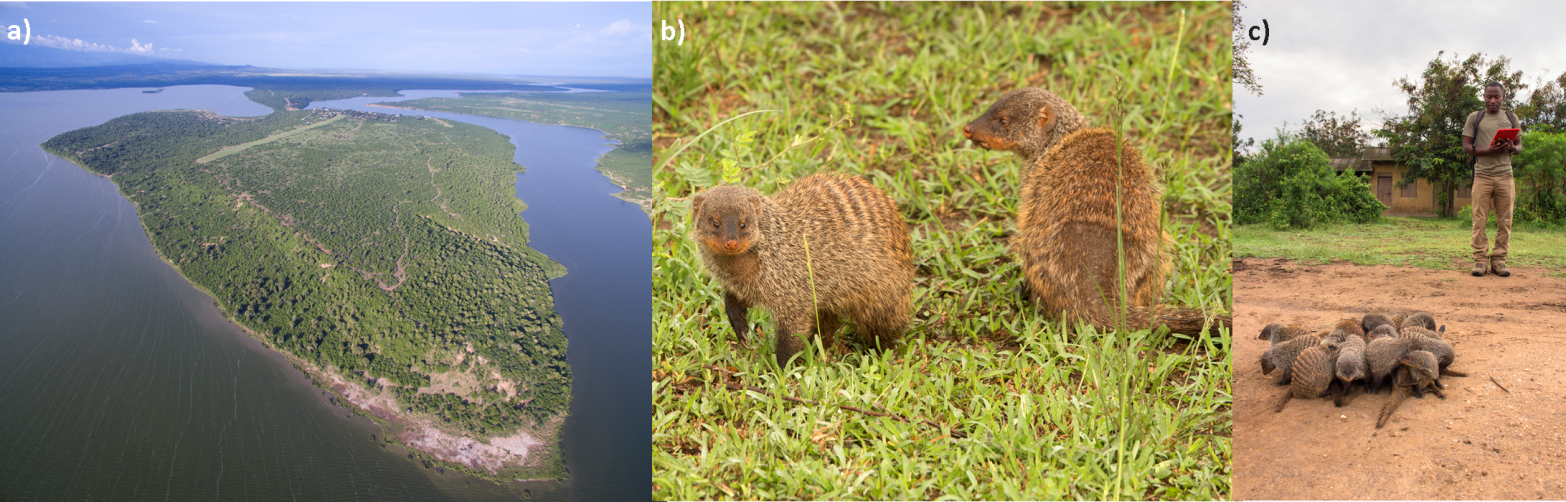
**The Banded Mongoose Research Project**: (a) the Mweya peninsula in Queen Elizabeth National Park, Uganda where the project is located; (b) banded mongooses, *Mungos mungo*; (c) one of the team of skilled fieldworkers (author SK) collecting data from a banded mongoose group. All photo credits: Feargus Cooney.

The Banded Mongoose Research Project (BMRP) is located on the Mweya Peninsula in Queen Elizabeth National Park, Uganda (central point: 0°11.5’S, 29°54.0’E; Fig 1A). The banded mongooses (*Mungos mungo*; Fig 1B) at this site were first studied in the 1970s [14,15] and have been studied continuously since 1995. Banded mongooses live in stable multi-male, multi-female groups of typically 10–30 individuals. They are cooperative breeders. That is, offspring in the group receive care from other members of the group (called helpers) as well as their own parents. Understanding why and how such helping behaviour evolved is a central question in ecological and evolutionary research, and provides one of the major motivations for the existence of the BMRP and many other long-term research projects [16]. Comprehensive descriptions of the BMRP study site and banded mongoose biology can be found in [17,18]. Here we provide a brief overview of the project’s data collection procedures and then outline the data collection and storage challenges this creates. All research procedures received prior approval from Uganda Wildlife Authority and Uganda National Council for Science and Technology, and adhered to the Guidelines for the Treatment of Animals in Behavioural Research and Teaching, published by the Association for the Study of Animal Behaviour. All research was approved by the Ethical Review Committee of the University of Exeter. We also confirm that author Solomon Kyabulima has completed and signed a consent form for an image of him to be used in Fig 1C.

The mongoose population at Mweya usually consists of around 250 individuals in 10 to 12 social groups, with 3,000+ individuals from 31 groups recorded by the BMRP over the past 20 years. All mongooses are individually marked using either unique hair-shave patterns or color-coded collars, and are habituated to observation from 3–20 metres by a team of skilled fieldworkers (authors FM, SK, KN and RB; Fig 1C). One to two mongooses in each group are fitted with a radio collar weighing 26 to 30 g (Sirtrack Ltd, Havelock North, New Zealand) with 20 cm whip antenna (Biotrack Ltd, Dorset, UK) to allow the groups to be located. Data collection involves visiting each mongoose group every 1–3 days for at least 20 minutes. During these visits the following data are recorded:

- Basic demography. This includes the presence or absence of individuals in the group (signifying death or dispersal), signs of pregnancy in the females and the occurrence of births.
- Weights. Once a week individuals are weighed in the morning before foraging starts and again in the evening after foraging has finished.
- Behaviour. Two forms of behavioural data are recorded. The first involves recording whether an individual was engaging in a particular behaviour during a group visit. These behaviours include: (1) individuals staying behind at the group’s den and ‘babysitting’ the pups during their first 30 days. This can be differentiated from absence due to death due its temporary nature (maximum 1 day) during a period when pups are known to be in the den, and absence due to dispersal as mongooses disperse in groups rather than singly [19]; (2) individuals caring for pups once they are out of the den and moving with the group by ‘escorting’ them; (3) males mate-guarding females when they are in oestrus, and so blocking other males’ access. The second form of behavioural data involves more detailed focal follows [20] of specific individuals during selected life stages. During these focal follows the identity of the nearest and other individuals within 2 m of the focal individual are recorded every minute. The occurrence of aggressive, affiliative and helping interactions between the focal and a second individual are also recorded.
- Location. The GPS location is recorded at the start and end of each group visit.

**Fig 2:**
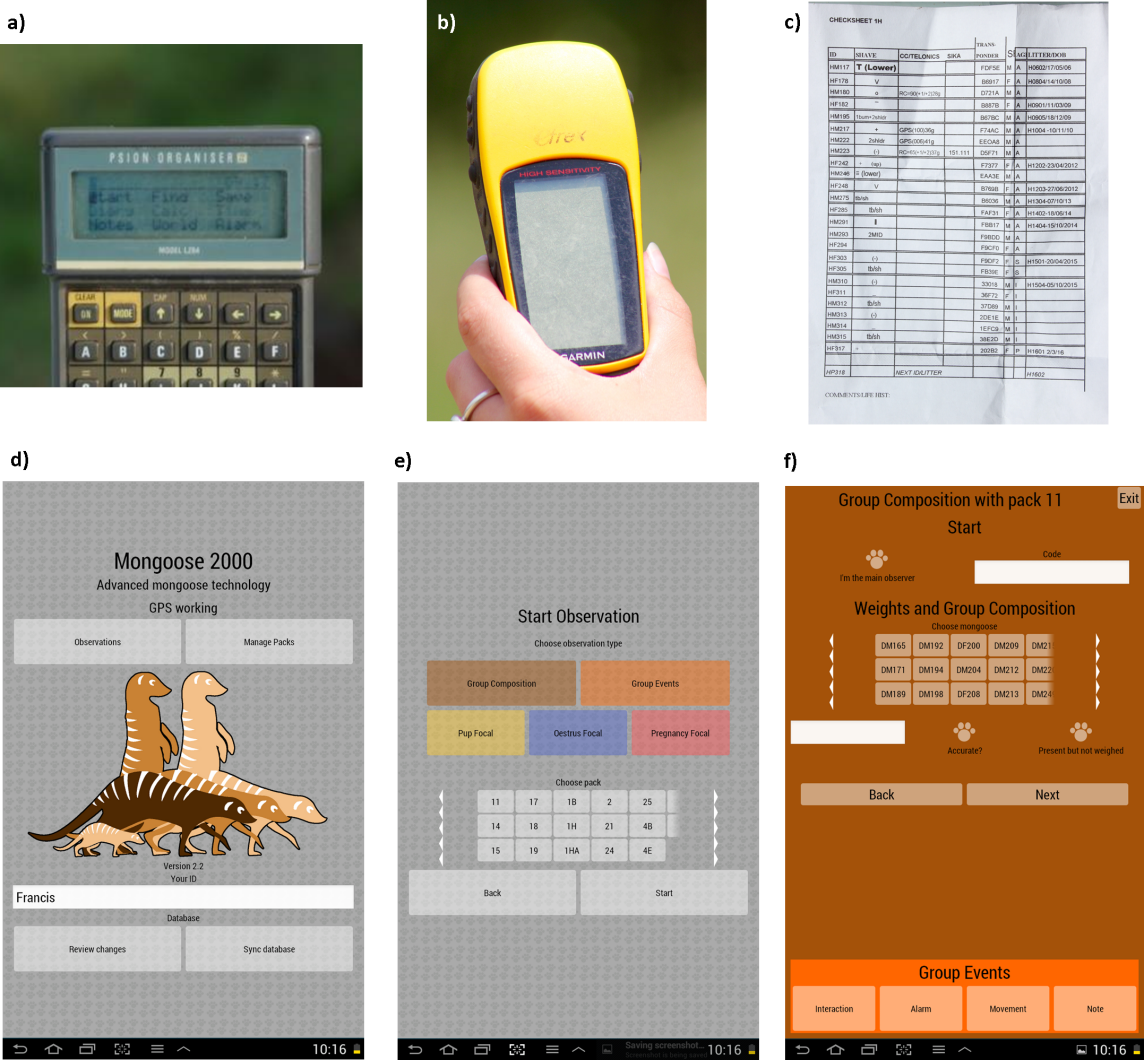
Past and present data collection interfaces. The handheld devices interfaces and checksheets used by the Banded Mongoose Research Project previously (a-c) and in the Mongoose 2000 system (d-f). Panel a: Psion II data logger, model LZ; b: handheld Garmin eTrex GPS unit; c: example of a paper checksheet, here listing all the individuals present in group 1H on a given day.

Until recently, these data were collected using a combination of handheld Psion II data loggers (model LZ), handheld GPS units (Garmin eTrex) and paper checksheets (Fig 2A–2C, respectively). These checksheets and handheld devices were then transported back to the project office where the data they contained were manually transferred into a central database on a computer. This general set-up and data transfer procedure is one that will be familiar to many field biologists. However, in our–and we suspect many other biologists'—experience it does have some drawbacks:

1. It is time-consuming. Transferring data from paper records to a computer, or from files stored on various hand-held devices to a computer can take a considerable time, particularly where this process requires the manual transcribing or processing of data before it can be incorporated into the central database [8]. It also requires some familiarity with the study system and the various software being used meaning it needs to be performed by skilled fieldworkers who could otherwise be using their time to collect data.
2. It is error-prone. Long-term, individual-based projects involve collecting a considerable number of records (often into the hundreds of thousands or millions). For example, the BMRP has 156,000 records of mongoose weights. Having to manually transcribe each of these records, often multiple times, inevitably leads to errors. For example, a simple typo can easily change the identity of a mongoose being weighed from individual 789 to 799 or its recorded weight from 1234 grams to 1334 grams. In subsequent checks of our data we estimate we find errors in about 5% of records.
3. There is a time lag between data being collected and being entered into the central database. Data collected in the field often stays on paper or stored on hand-held devices for a considerable time (i.e. at least a number of days) until the fieldwork schedule allows time for a fieldworker to input the data into the central database. This lag potentially allows further errors since any query over the raw data collected becomes more difficult to assess the longer it has been since the observation was made. This time lag also inhibits information sharing about recent events in the study system between fieldworkers. Fieldworkers cannot simply interrogate the central database for the latest information but need to talk to each other. Breakdowns in this communication can lead to errors such as the same data being recorded twice (e.g. the occurrence of a birth or death) or data being recorded using out of date information (e.g. an individual being recorded as a babysitter when they were observed dead the previous day). This is less problematic in small team of fieldworkers (e.g. 1–2 people) but rapidly becomes more problematic as the team expands.
4. Existing hand-held devices are outdated with limiting interfaces. This last issue will depend on the particular technology being used by a long-term project, however, we believe that many projects use the same hand-held devices for many years. These devices inevitably become outdated and have limiting interfaces compared to what current technology offers (Fig 2). When these handheld devices were first developed they provided considerable improvements in data collection efficiency and accuracy, however, they have now been superseded by more powerful and versatile smartphones and tablet computers. The limited interfaces they offer often require data collection protocols to be modified (e.g. using abbreviated versions of individual ID codes) and their use is often not intuitive meaning fieldworkers need particular training in their use before they can effectively collect data.

## The Mongoose 2000 system

To address the data collection and storage challenges we outline above, the BMRP formed a collaboration with FoAM Kernow (http://fo.am/kernow/), a non-profit organisation that develops links between computer technology, science and design. The aim of this collaboration was to develop a system that utilised the potential of apps and portable computers to collect our data in a more effective and efficient manner. In this section we describe this system, affectionately named Mongoose 2000. We then discuss in the final section how this system has improved the BMRP data collection and storage, where it still has limitations, how we plan to develop it in the future and how other long-term individual-based projects could potentially benefit from using a similar system. The source code needed to build the system is freely available under open-source GPL license. The current release’s source code (version 2.5 at the time of writing) is available from http://dx.doi.org/10.5281/zenodo.55222 and further updates can be found on GitHub at https://github.com/nebogeo/mongoose-web.

### Platform requirements and availability

The Mongoose 2000 app is designed for all versions of the Android operating system. It is designed with a tablet computer screen size in mind since tablet computers meet our logistical needs at the BMRP. However, the app can also be used on mobile smartphones running the Android operating system (e.g. by adjusting the text size in the device’s settings). The app can be installed via the Google Play Store: https://play.google.com/store/apps/details?id=foam.mongoose. The server runs on a Raspberry Pi version 1B, on the standard Raspbian OS.

### Hardware and programming

**Fig 3:**
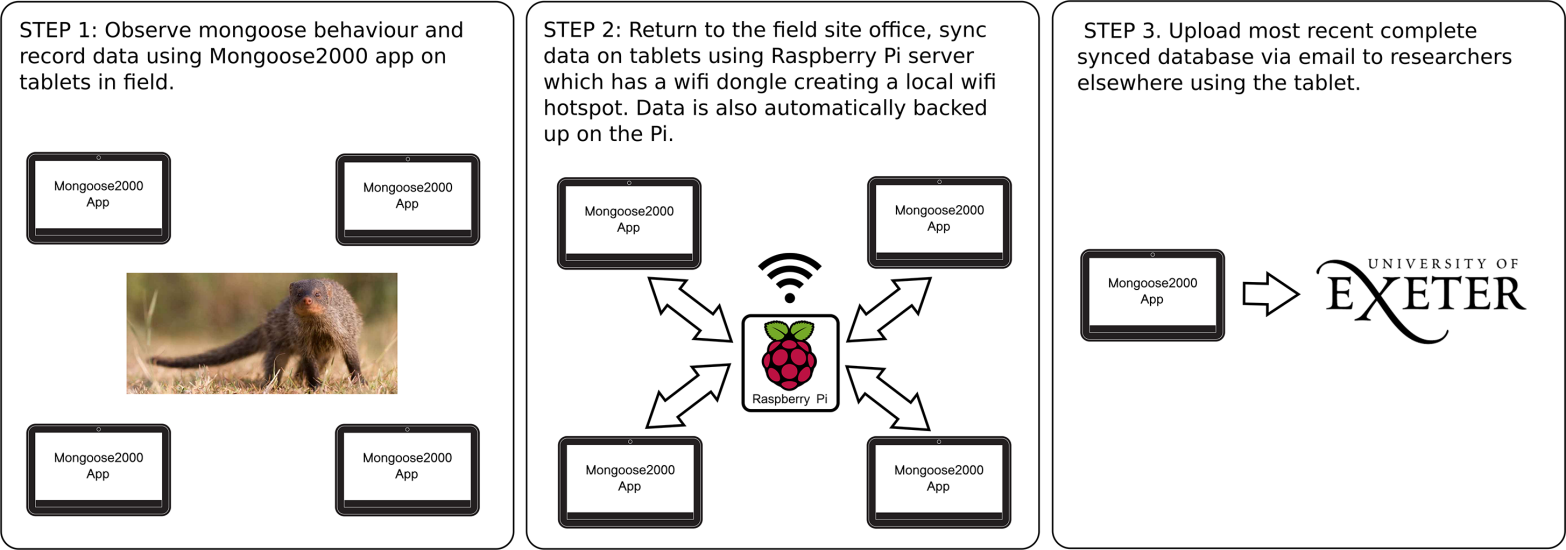
A schematic of the Mongoose 2000 system. Data are collected at the field site using Android tablets. When fieldworkers return to the field site office they connect the tablets to a Raspberry Pi server running a local Wi-Fi network (Mongoose-web). The tablets synchronise their data to this central server and are also able to download the entire database and email it to UK-based mongoose researchers at the University of Exeter (or any other external location).

The system comprises two parts, a server running on a single Raspberry Pi equipped with a USB Wi-Fi dongle that provides a local hotspot, and multiple android devices (we use Samsung Galaxy Note 10.1 tablets) running the app on which the data are collected (Fig 3). No internet connection is required, as the system runs entirely on its own network. When switched into synchronisation mode the tablets search for the Mongoose-web Wi-Fi SSID presence and connect with the Raspberry Pi in order to upload new data to the server and download any new data about the mongoose population (see details in ‘Data storage’ section below).

The application and server software is written in Scheme, and the core synchronisation functionality is provided by the same code both on Android and Raspberry Pi for consistency. On the Raspberry Pi the server code is running on Racket (Servet HTTP server) while on the Android the main code is running in a TinyScheme interpreter compiled using the Android native development kit (C++) passing interface updates to the Android SDK in Java. Both the server and app clients use SQLite3 for persistent database storage of synchronised data.

The interface design for Mongoose 2000 (Fig 2D–2F) had to take into account the long running, continuous nature of the research. Designing a system to be used every day for the next 10 years is an unusual use case in comparison to most types of commercial app design. Care was taken to provide all interface elements on a single screen, with no scrolling required. Features such as consistent positions of individual mongoose IDs to enable user 'muscle memory' to build up had to take precedence over the kind of simplicity usually needed for apps that are used infrequently.

### Field user walkthrough

The Mongoose 2000 app presents the user with four buttons when first opened: ‘Observations’, ‘Manage packs’, ‘Review changes’ and ‘Sync database’. These represent the app’s four modes. Here we briefly describe the operations that each mode carries out.

#### Observations

This contains Mongoose 2000’s data collection functions. Once users select this mode they are asked to select the mongoose group on which they are collecting data, and to select between three different types of data collection: ‘Group Composition’, ‘Focal Follows’ and ‘Group Events’. Selecting each starts a sequence of screens allowing the user to input the relevant data types (see Table 1).

**Table 1:** The data recorded in each of the three different screen sequences available in Mongoose 2000’s Observations mode.

Group composition	Focal follows	Group events
Presence/absence from the group	Scans every minute - identity of nearest neighbour - identity of all individuals within 2 m	Group-level events such as: - interactions with other groups - alarms at potential threats - group movement decisions
Weight		
Female pregnancy status (binary)		
Mate-guarding - identity of male and female -strength of the association (qualitative score) -accuracy of data (qualitative score) -male is primary guard? (binary)	Aggressive and affiliative interactions with other individuals -identity of other individual -who initiated the interaction -further information such as the intensity of the interaction (e.g. pushing, attacking), its context (e.g. food, social partner) and who won the interaction (e.g. in an aggressive encounter)	Information that can be recorded with each includes: -the identity of individuals involved -the outcome of the group interaction (e.g. which group retreated) -the cause of the alarm (e.g. sighting a predator)
Pup escorting -identity of pup and escort -strength of the association (qualitative score) -accuracy of data (qualitative score)		
Babysitting -identity of individual -whether directly observed or inferred by temporary absence from the group		
Start and end time of group visit		
GPS location of group every 3 minutes		

Group composition. This screen sequence is started when fieldworkers arrive at any mongoose group. Starting the sequence logs the group visit start time and starts the automatic logging of the group’s GPS location every 3 minutes throughout the group visit. The user is initially presented with a screen where they can select which of the mongooses previously recorded as present in the group are present on this visit. They can also record each mongoose’s weight during the weekly group weighing visits. The information provided here about which mongooses are present, in combination with information held in the database about individuals’ age and sex (see Manage packs mode below), then determines which individuals are available for selection in the following screens. These screens allow the user to record data on which females are pregnant, which males are mate-guarding females, which pups are being escorted by which adults and which individuals are babysitting pups at the den (Table 1). Where it is not appropriate to record these data (e.g. because there are no pups in the den to babysit) the user can signify this by skipping the screen.

Focal follows. The focal follow screen sequences allow the user to collect focal follow data (Altmann, 1974) from specific individuals. In the Mongoose 2000 app there are separate focal follow protocols for three groups of individuals: dependent pups, pregnant females and females in oestrus. Data collection from each of these three groups is driven by the Banded Mongoose Research Project’s current research questions. The structure of all the focal follow protocols is the same, as is the app screen sequence. After selecting a particular focal follow protocol in the Mongoose 2000 app the user is then asked to select the identity of the focal individual. This starts a 20 minute countdown. Each minute the user is prompted to record the identity of the focal individual’s nearest neighbour and identity of all individuals within 2 metres of them. During the 20 minutes a series of buttons is available to the user allowing them to record the occurrence of any affiliative or agonistic interactions the focal individual has with other group members. When one of these buttons is selected the app then prompts the user to input further information about the interaction, such as the identity of the other individual involved, the direction of the interaction, the context of the interaction (e.g. over food or a social partner) and further details about the type of affiliative or agonistic interaction (e.g. for agonistic interactions whether it was one individual body-blocking or attacking another). The 20 minute countdown can be paused at any time, such as when the focal individual temporarily goes out of sight from the user, and can also be exited completely if the focal follow is terminated early.

Group events. The group events screen sequence allows the user to record group-level events such as interactions with other groups, group movement decisions and alarms at threatening stimuli. It is available for the user to select directly from the initial screen in the Observations mode but is also available to select directly from the group composition and focal follow sequences to allow these events to be easily recorded ad hoc whilst other data are being collected from the group. When a particular event is selected the user is then prompted to provide more information about it, such as the identity of the individuals or other group involved, the stimulus that caused the alarm, or the particular feature that the group movement decision was orientated towards or away from (e.g. a water source or den site). The group events options also contain a ‘Note’ button where the user can record any free text notes to support their observations, such as errors in data collection to be rectified later (see Review Changes mode below).

#### Manage packs

This mode allows the user to manage which individuals are recorded in which groups, and information about these individuals such as their birth date and sex. The user is initially shown all the groups in the database. Selecting a group then shows all the individuals recorded in the group, and selecting an individual displays the information recorded about them. Options are available to add or remove groups (as new groups enter the study area or groups die out) and add or remove individuals who are born/die or immigrate into/emigrate out of the population.

This information is used by the three screen sequences in the Observations mode (see above) to determine which individuals are available to select in a particular data collection screen. For example, in the Group Composition sequences when prompting the user to select which females are pregnant the app will only make the identities of the adult females (> 10 months old) in the focal group available for selection.

#### Review changes

This mode allows users to review and edit the data they have recorded in the Observations mode. Its primary purpose is to allow users to correct any data collection errors before they are sent to the central server (Fig 3, see Sync database mode below and Data Storage section next). Users are presented with a list of each type of data they have collected during each group visit, for example a group composition session on group A on 11/5/16. Selecting these then displays each observation recorded under that heading, for example all the records of presence/absence. Users can then select these records and edit them, for example changing an individual being recorded as absent to present.

#### Sync database

This mode allows the user to synchronise the data they have collected with the central database, receive any changes made to the group lists by other users and also download the entire database from the central node for sending elsewhere. Once users are in range of the Raspberry Pi server, users simply have to select the ‘Sync me’ or ‘Download’ buttons and the app automatically connects the server and uploads or downloads the data.

### Data storage

All data is stored internally using the SQLite database. This database type was chosen due to the low computational capabilities of both the Raspberry Pi and the Android tablets, and the high reliability and maturity of this open source software. The database is stored in a single binary file. This simplifies backup procedures as it simply requires the emailing of this file from the tablet as an email attachment.

The synchronisation process is two way. First, new entries or modifications in the data stored on the tablet are marked automatically as needing to be synced as they are edited or added by the users. When the tablet is put into synchronisation mode it sends each one of these changes to the server and awaits confirmation that it was received, and marks the entry as synced. When the server receives a new entry it updates it to the new value and increments a version number associated with every entry. Second, when the app has sent all of the new entries to the server it then sends the server a list of version numbers from its local database. The server then sends back any entries it has that are newer, for example changes made to the group lists by other users.

The synchronisation is designed to work when multiple tablets are connecting simultaneously, so entries will be updated in interleaved order, some tablets sending while others receive. As a general rule, if a conflict emerges (two changes to the same entry occur at the same time) the last tablet to sync will take priority. As entries consist of a single element of data in a larger object (e.g. the date of birth of a mongoose is one entry), conflicts are rare.

A further consideration, due to the nature of the research project, was flexibility to incorporate future changes to data collection protocols. The precise form of the data collected needed to be changed and updated continuously, both during the development process as we added new observations, and also as the needs of the research changed (for example the addition of GPS data). The Raspberry Pi software is stored on an SD card in the field and not connected to the internet, therefore updating this is problematic (requiring a postal service). The tablets on the other hand are easy to update online via the normal Play Store update mechanism, so the server had to cope with new data types and entire new observation categories suddenly appearing. To support this the app utilises an entity-attribute-value system [21], so new table columns can be added without a database schema change on the Raspberry Pi server.

Data are downloaded by emailing the SQLite database file from the tablets at the field station. Each tablet contains its own version of the data, and so each tablet acts as a backup in case of damage to the server. Backups are taken on a weekly basis and the database is sent to the UK-based project team at the University of Exeter on a monthly basis. To ensure these weekly tablet backups are remembered each tablet is assigned to a particular fieldworker who has responsibility for its backups. Similarly, one person in Uganda is assigned the role of sending the monthly data emails to a designated person on the UK. These regular activities are supported by reminders from a shared Google calendar and a paper schedule on the field office wall. For security, data are never deleted, so continuously build up over time. Currently we clear the system (replace the SD card and reset the tablets) on a six month basis. After a reset, persistent data such as group and individual data are automatically retained.

When received from the field, the SQLite files are processed by a Python application (http://dx.doi.org/10.5281/zenodo.55221) which allows the researchers to select data types and export them as CSV files for further analyses. These analyses are used to answer evolutionary and ecological research questions (e.g. [22–24]) and data supporting them made available in online repositories such as Figshare and data dryad.

## Discussion

In this paper we have introduced the Mongoose 2000 system as a case study of how developing apps for use on portable computers can allow researchers working on long-term, individual-based ecological projects to efficiently collect and store data in the field from multiple sources. Here we discuss some of the benefits and limitations we have experienced in using this system, how the system may be developed in the future and its potential for use on other research projects.

### Benefits of the Mongoose 2000 system

The immediate benefits of the Mongoose 2000 system are that it addresses the four limitations we identified in our project’s (and other’s we suspect) previous data collection system that utilised multiple hand-held devices and paper checksheets from which data was being manually transferred to a central database. Specifically, the system addresses these limitations as follows.

1. *It was time-consuming*. The main time saving feature of Mongoose 2000 is the automated synchronisation of data to the central database. Previously, transferring each day’s data to the central database manually took hours of a fieldworker’s time whereas the automated system on Mongoose 2000 allows this process to happen in a few minutes. In addition, the Mongoose 2000 system makes field data collection much more efficient by allowing collection of many data types on one device rather than several, often automatically (e.g. GPS locations).
2. *It was error-prone*. The automated synchronisation of data to the central database removes all errors from manually transcribing the data incorrectly. This improvement is illustrated by a comparison of the error rate in our weight records collected using the Mongoose 2000 system compared to our previous system. In 720 weight records collected on the Mongoose 2000 system between November 2014 and March 2015 during field tests we found a 2% error rate (18 out of 720 records that were not feasible given the age and sex of the mongoose). This is an improvement on the 5% error rate we estimated in the old system and is likely to come from these data only being manually inputted once (on first collection) rather than multiple times as before (e.g. first collecting the data and then manually transcribing the data into the project database). In addition the Mongoose 2000 system has additional features which are likely to reduce this error further. The synchronisation of the list of individuals present in each group across all fieldworkers’ tablets means that each mongoose identity only has to be input/removed once into/from the system rather than multiple times on each hand-held device. This significantly reduces the possibility of an identity being incorrectly recorded due to a typo. The Mongoose 2000 app also contains various internal checks to further cut out incorrect data collection, e.g. in the group composition screen sequence whether a mongoose is marked as present in the first screen determines what data can be collected about them in later screens. The remaining 2% error rate predominantly comes from typos when entering in values in to free text fields, e.g. weight values. This rate could be further reduced in the future by incorporating further internal checks of the feasibility of the entered value, e.g. checking weight records against previously recorded values for that individual and against a range of realistic values given the individual’s age and sex.
3. *There was a time lag between data being collected and it being entered into the central database*. The ability to synchronise data to the central database means that Mongoose 2000 field users can error check their field data as soon as they get back to the office in a process usually taking about twenty minutes per day (rather than data taking days to be entered into the central database in our previous system). This shorter time lag has made it easier to identify any potential errors in field data collection and communicate important observations (e.g. births and deaths) between field workers.
4. *Existing hand-held devices were outdated with limiting interfaces*. The use of modern tablet computers has allowed the collection of many different data types to be consolidated onto one hand-held device and a considerable improvement in computing power and memory compared to the previous devices we used. Any new computing technology is almost certain to become superseded by a newer technology in the future. However, we hope that we have maximised the time it will take for the Mongoose 2000 system to be superseded by making it open source and designed to run on the widely used and open-source Android operating system. Fig 2 also illustrates the differences between the interfaces on the previous hand-held devices and in Mongoose 2000 on a tablet computer. By being much larger and in colour tablet (and smartphone) interfaces allow a far more flexible and intuitive display. They also utilise touchscreen buttons rather than a keyboard for data input. Both of these features make the training of fieldworkers to use the app considerably easier and quicker and also further reduce data collection error. Another example of a field data collection app that has used intuitive touchscreen interfaces to great effect is Cybertracker (cybertracker.org).

In addition to these four immediate benefits, the Mongoose 2000 system has some broader advantages over other field data collection apps and programs that have looked to take advantage of portable computing technology such as tablets and smartphones. First, the Mongoose 2000 system requires no external networking. Instead, the Raspberry Pi server creates its own local wireless network to which the tablet computers (or smartphones) all connect to upload and synchronise data (Fig 3). Therefore, unlike in other data collection apps (e.g. EpiCollect [8]), data upload and collation across multiple devices in the Mongoose 2000 system is not reliant on mobile phone or internet connectivity, which can often be unreliable or non-existent at many remote field locations. This means that data can be quickly and regularly shared across multiple devices (for example, changes in mongoose group membership in our case) and that data are held in multiple locations as insurance against the loss or damage of one of the devices. Second, the system is open source, with all the source code needed to build it freely available and new versions available for download for free from the Google Play Store (see links in section 3). This is unlike other field data collection programs such as CyberTracker (cybertracker.org). As a result other field biologists can fully customise the system to fit their own data collection needs. There are currently some limitations to this ability, but also some future developments we hope to implement to address these limitations, which we discuss in the final section.

### Limitations of the Mongoose 2000 system

Despite the significant benefits of the Mongoose 2000 system detailed above there are also some limitations. Although we have made the system open source, negating the need to develop it from scratch, reasonably advanced programming knowledge is needed for other researchers to customise it for their own data collection requirements (compared to programs such as Cybertracker, cybertracker.org, or EpiCollect [8]). It is likely that, with the increasing use of portable computers and apps, such knowledge will become increasingly common in non-specialists. For researchers without this specialist programming knowledge there are other options available such as collaborating with a computer scientist (see further discussion in [9]). If options such as collaboration are not possible then researchers may need to hire a professional app developer. Developing the Mongoose 2000 system to this point involved around 70 days’ work by a non-profit developer at around £220/day. The amount of time needed to customise the system to another similar research project will depend on the project’s requirements, but we estimate 10–15 days’ developer time would be sufficient. Therefore, where advanced programming knowledge is not available to researchers from within their research group or through collaboration they may be limited in their ability to use the Mongoose 2000 system until they can apply for funds to pay a developer. However, as we describe in the next section, we are hoping to develop the system further to relax this limitation.

In addition to these potential development costs, there are also hardware costs to using the Mongoose 2000 system. However, we suspect these are manageable for most long-term research projects. These include the cost of tablets or smartphones running the Android operating system and the Raspberry Pi server. We use Samsung Galaxy Note 10.1 tablets which are high-spec models chosen to maximise the time before they became obsolete and cost £365 each in 2015. Researchers working in more extreme environments (e.g. hotter or wetter) may need more expensive devices that are, for example, waterproof or shockproof. In our experience, however, there are low-cost alternatives to protecting portable computing equipment in all but the most challenging environments (e.g. Otterbox covers, otterbox.co.uk, for ~£50 used to protect standard handheld devices in [25]). In addition, there are cheaper options to the portable devices we used. The Mongoose 2000 system will run on any portable device running the Android operating system and, for example, tablets running this are available for ~£50. Furthermore, like any computing technology, hardware costs are highly likely to fall in the future. The Raspberry Pi with Wi-Fi dongle cost £35.

Further limitations in the use of the Mongoose 2000 system revolve around the time investment required. Whilst Mongoose 2000 saves considerable time in field data collection and storage (see above) it requires a time investment upfront to design the system in collaboration with developers, and to trial the system in the office and then in the field. In our case from the first discussions about the system to first trialling it at the field site has taken around 2 years involving around 5 hours of a researcher’s time per month (plus the developer’s time). This time requirement is likely to be halved to around 1 year by researchers customising Mongoose 2000 system to their own requirements rather than starting from scratch. The time period could of course be further shortened by a greater per month time investment by the researcher. There was a further 6 month period when the Mongoose 2000 system was run in parallel with the old system to check if it was collecting comparable data and to fix any issues that field testing raised. This data checking period is conservative but probably prudent for any research project switching from one data collection system to another (though note it would not be needed for a project starting in a system with no previous data collection). In total, this produces an estimate of 1 to 1.5 years (depending on researchers’ per month time commitment) for researchers to customise and implement a data collection system based on Mongoose 2000 in their own research project. Whilst not inconsiderable this investment is less than the standard 3 year length of a studentship or grant, and involves time investment from a researcher spread thinly over the period. As a result it should be feasible for most research projects to implement such a data collection system by writing its development into a larger grant application, applying for smaller grants specifically for the work or by tasking current project researchers to its implementation without significantly impacting their other responsibilities. In addition, we hope that further developments to the system (see next section) will make it more easily customisable and further reduce the time investment discussed here.

### Future development and broader uses

In the future we plan to develop the Mongoose 2000 system in two particular ways. First, we plan to develop a laptop/desktop computer based interface for the app. This interface will include integrating the database held on the Raspberry Pi server with an SQL-based database on a computer allowing the data recorded on the app to be easily viewed and extracted for analyses. It will also include a component that will allow the addition and customisation of the data collection functions in the app by users without advanced programming skills. We hope this will negate some of the time and expense limitations associated with the need for advanced programming skills that we discuss above. Second, we also plan to develop the capability to record life history events such as births and deaths, and immigrations into and emigrations out of the study groups or population. Currently it is possible to add or remove individuals from the list of each group’s members. However, full life history event data collection (i.e. the reasons for the additions and removals) is not implemented as some events require retrospective use of data from previous days’ data collection. For example, we only confirm a mongoose as having died if they have been consistently absent from the population for more than three days (unless we find physical evidence of death, but this is rare). Such internal checks are important in the accurate collection of life history (or any) data and in the future we plan to include the ability to perform these checks through the retrospective use of previous days’ data.

This summary of the current Mongoose 2000 system demonstrates the potential uses of apps and portable computers for data collection in long-term individual-based ecological and evolutionary research projects. In particular their use offers considerable advantages in terms of time saved and reduced data collection errors. Currently, the system is specific to our banded mongoose study system and other research projects may be limited in their ability to develop similar systems by the time and financial investment required. However, we hope that the further developments that we plan to make to the Mongoose 2000 system, particularly the ability to customise the data collection functions without advanced programming skills, will negate many of these limitations and make it usable by many long-term research projects. Since the code is available open-source, other research groups can freely build upon this work for their own purposes.
